# Do Participants’ Preferences for Mode of Delivery (Text, Video, or Both) Influence the Effectiveness of a Web-Based Physical Activity Intervention?

**DOI:** 10.2196/jmir.1998

**Published:** 2012-02-29

**Authors:** Corneel Vandelanotte, Mitch J Duncan, Ronald C Plotnikoff, W Kerry Mummery

**Affiliations:** ^1^Centre for Physical Activity StudiesInstitute for Health and Social Science ResearchCentral Queensland UniversityRockhamptonAustralia; ^2^Priority Research Centre in Physical Activity and NutritionSchool of EducationUniversity of NewcastleNewcastleAustralia; ^3^Faculty of Physical Education and RecreationUniversity of AlbertaEdmonton, ABCanada

**Keywords:** Physical activity: computer tailoring, mismatch, preferences, delivery method, website-delivered intervention, behavior-change intervention

## Abstract

**Background:**

In randomized controlled trials, participants cannot choose their preferred intervention delivery mode and thus might refuse to participate or not engage fully if assigned to a nonpreferred group. This might underestimate the true effectiveness of behavior-change interventions.

**Objective:**

To examine whether receiving interventions either matched or mismatched with participants’ preferred delivery mode would influence effectiveness of a Web-based physical activity intervention.

**Methods:**

Adults (n = 863), recruited via email, were randomly assigned to one of three intervention delivery modes (text based, video based, or combined) and received fully automated, Internet-delivered personal advice about physical activity. Personalized intervention content, based on the theory of planned behavior and stages of change concept, was identical across groups. Online, self-assessed questionnaires measuring physical activity were completed at baseline, 1 week, and 1 month. Physical activity advice acceptability and website usability were assessed at 1 week. Before randomization, participants were asked which delivery mode they preferred, to categorize them as matched or mismatched. Time spent on the website was measured throughout the intervention. We applied intention-to-treat, repeated-measures analyses of covariance to assess group differences.

**Results:**

Attrition was high (575/863, 66.6%), though equal between groups (*t*_86__3_ =1.31, *P* =.19). At 1-month follow-up, 93 participants were categorized as matched and 195 as mismatched. They preferred text mode (493/803, 61.4%) over combined (216/803, 26.9%) and video modes (94/803, 11.7%). After the intervention, 20% (26/132) of matched-group participants and 34% (96/282) in the mismatched group changed their delivery mode preference. Time effects were significant for all physical activity outcomes (total physical activity: *F*_2,801_ = 5.07, *P* = .009; number of activity sessions: *F*_2,801_ = 7.52, *P* < .001; walking: *F*_2,801_ = 8.32, *P* < .001; moderate physical activity: *F*_2,801_ = 9.53, *P* < .001; and vigorous physical activity: *F*_2,801_ = 6.04, *P* = .002), indicating that physical activity increased over time for both matched and mismatched groups. Matched-group participants improved physical activity outcomes slightly more than those in the mismatched group, but interaction effects were not significant. Physical activity advice acceptability (content scale: *t*_368_ = .10, *P* = .92; layout scale: *t*_368_ = 1.53, *P* = .12) and website usability (layout scale: *t*_426_ = .05, *P* = .96; ease of use scale: *t*_426_ = .21, *P* = .83) were generally high and did not differ between the matched and mismatched groups. The only significant difference (*t*_621_ = 2.16, *P* = .03) was in relation to total time spent on the website: the mismatched group spent significantly more time on the website (14.4 minutes) than the matched group (12.1 minutes).

**Conclusion:**

Participants’ preference regarding delivery mode may not significantly influence intervention outcomes. Consequently, allowing participants to choose their preferred delivery mode may not increase effectiveness of Web-based interventions.

## Introduction

Physical inactivity increases the risk of developing cardiovascular disease, diabetes, hypertension, some cancers, and obesity [1,2]. As large proportions of the population are not meeting physical activity guidelines [3-5], increasing physical activity is a public health priority. As such, intervention strategies that can reach many people in a cost-effective manner are desired.

Web-based physical activity interventions have shown promising results [6-8] and will continue to gain importance through growth in Internet access (in Australia, 73% of households have broadband access), the power of Web-based applications (eg, Facebook or YouTube), and convenience (through mobile devices) [9,10]. However, the immense versatility of the Internet allows for health information to be delivered in several ways [8]. For example, interventions delivered via websites can provide personally tailored information through different modes, such as text based, video based, or both [11]. While personally tailored interventions have been shown to be effective both in offline (print based) and online studies [12-15], there are large variations in individual preferences for the mode of intervention delivery [16,17]. This raises the question as to whether the effectiveness of an intervention may be enhanced or reduced when it is provided through a preferred or nonpreferred mode [18].

This may be important, as a review of randomized controlled trials found that a substantial proportion of potential participants refused to participate in these trials for fear of being assigned to the nonpreferred option [19]. Participants may also drop out of a trial after being assigned to the nonpreferred mode, or may enter and remain in the study but not adhere to or be engaged in the treatment [20]. As such, randomly assigning participants to nonpreferred delivery modes may reduce their participation, follow-up, and satisfaction, and may thus lead to poor outcomes [21]. Conversely, allocation to the preferred intervention delivery mode may lead to greater participation and better intervention effectiveness [21]. Ideally, however, population-based interventions should be robust and optimally effective no matter which delivery mode participants prefer.

Numerous studies have compared the effectiveness of different intervention delivery modes [22-26], but few have evaluated intervention effectiveness when participants were matched with their preferred intervention delivery mode. This is because randomized controlled trials are the gold standard in intervention research, so participants do not get to choose their preferred mode of delivery [27]. Therefore, the effect that delivery mode preferences may have on intervention outcomes remains largely unknown [28]. It could be argued that randomized controlled trials underestimate the gains possible in real-life intervention implementation [29], simply because no effort is made to match delivery mode with preference.

To our knowledge, only one behavioral study has examined whether preferred and nonpreferred modes of delivery influence intervention effectiveness. In a comparison of interventions delivered by print or telephone, Lewis et al [18] found that being in the preferred group did not influence physical activity levels. A few medical studies have also examined the influence of preferences on treatment outcomes, but with inconclusive results: some studies found better effects on satisfaction [20] and effectiveness [30] when participants were allocated to the preferred group and others not [19]. Overall, there is little evidence that preference effects significantly compromise internal validity [19], but this conclusion rests on a small number of studies. If preference has indeed little impact on effectiveness, the most cost-effective practice would be to develop only the delivery mode shown to have the greatest impact on health behavior, as the development of different intervention delivery modes is time consuming and costly. This might be more important for Web-based interventions where participants are simply given a choice to receive the intervention in a different way via the website, and where developers should avoid designing costly alternative delivery modes if they will not make a substantial difference.

Nevertheless, we need to know what works for whom; as such, there is a need for algorithms that will efficiently and effectively match participants to physical activity programs that best meet their needs [31]. Computer-tailored interventions aim to do this by providing information that is as personally relevant as possible [32]. However, as alluded to above, it is perhaps not only the content of the health information that needs to be tailored to the individual to achieve optimal effectiveness [13], but also the mode by which the intervention is delivered. Therefore, this study aimed to examine the impact of receiving computer-tailored intervention content that is either matched or mismatched with participants’ preferred mode of delivery (text based, video based, or both) on the acceptability, usability, and effectiveness of a Web-based physical activity intervention.

## Methods

### Design

We conducted a three-arm, randomized trial with balanced allocation ratio to assess the effectiveness of a Web-based, computer-tailored physical activity intervention. The intervention content was identical across the three arms; however, this content was delivered in a different mode for each group. The first arm received personalized physical activity feedback intervention in video mode, the second arm received the intervention in text mode, and the third arm received the intervention in combination mode, which provided both video- and text-tailored information. Data were collected during three measurement waves: baseline, 1-week follow-up, and 1-month follow-up.

### Participants and Procedure

**Figure 1:**
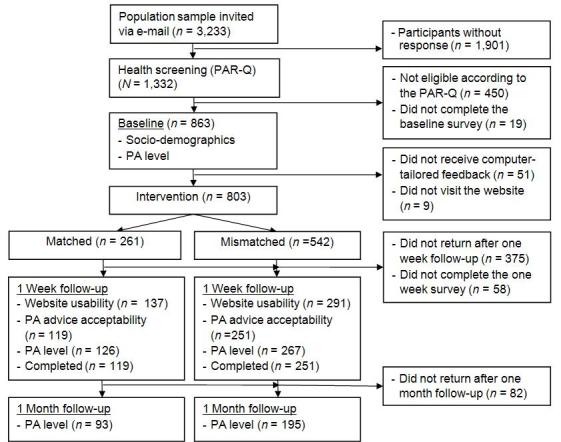
Participant flow. PA = physical activity; PAR-Q = Physical Activity Readiness Questionnaire.

In January and February 2011, male and female adults over 18 years of age from the general population in Australia were invited by email to participate in the study. People listed in a database held by the Population Research Laboratory at the Institute of Health and Social Sciences Research at the University of Central Queensland were invited. To be eligible, participants had to have Internet access and no medical constraints that would prevent an increase in physical activity. The invitation emails contained a link to a website with information about the nature and purpose of the present study and access to the baseline survey. By accessing the baseline survey, participants provided consent to participate and agreed that they were well informed about the study. We used the Physical Activity Readiness Questionnaire (PAR-Q) to screen for participants for whom it was not safe to increase physical activity [33]. If participants answered yes to one of the PAR-Q questions, they were thanked for their time and not provided with access to the intervention website. After completing the baseline survey, participants were given a link to the intervention website; participants were automatically randomly assigned to one of the three groups on accessing the website. Nonresponders were reminded three times to complete each assessment. The whole study was entirely Web-based without any face-to-face components as part of the intervention or the assessment; as such, real-life conditions were mimicked as closely as possible. The study received ethical approval by the Human Research Ethics Committees at Central Queensland University. Figure 1 provides an overview of participant flow.

### Intervention

**Figure 2:**
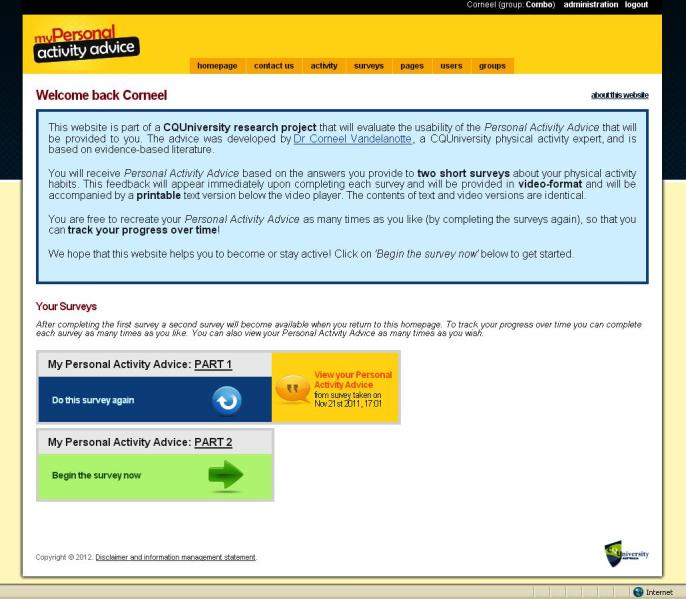
Screenshot of introduction/home page.

**Figure 3:**
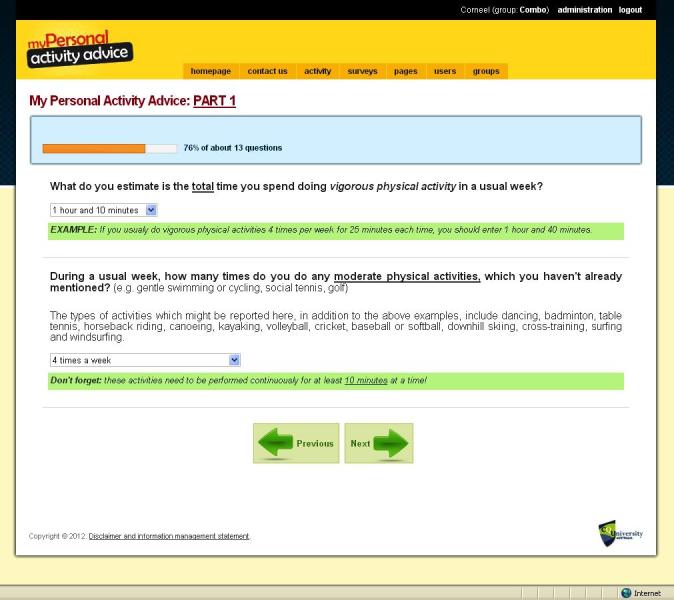
Screenshot of survey questions.

**Figure 4:**
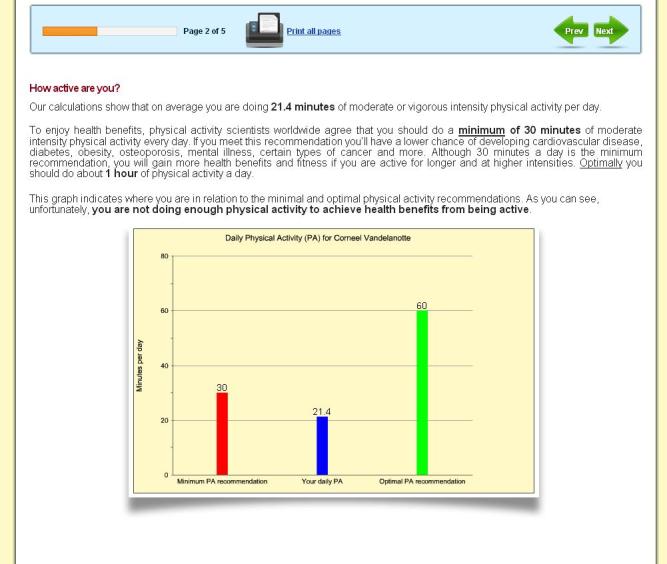
Screenshot of text mode feedback.

**Figure 5:**
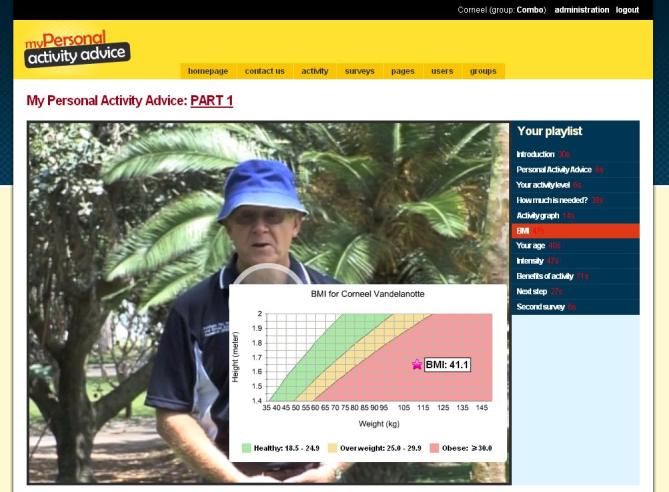
Screenshot of video mode feedback.

**Figure 6:**
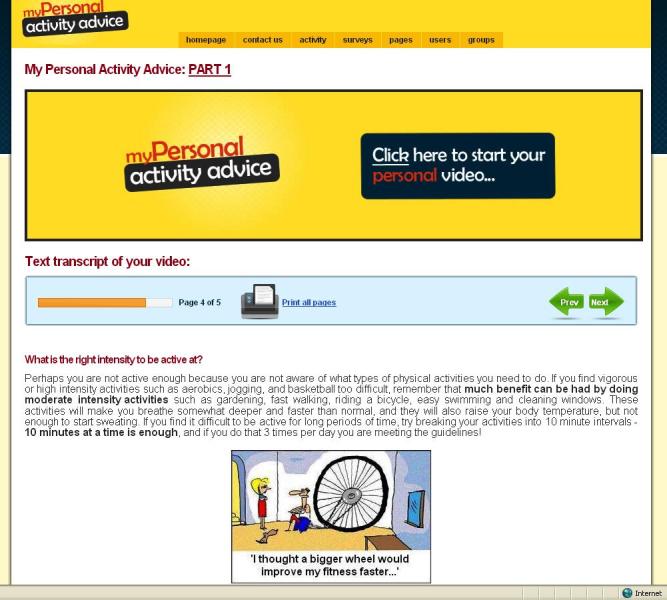
Screenshot of combination mode feedback 1.

**Figure 7:**
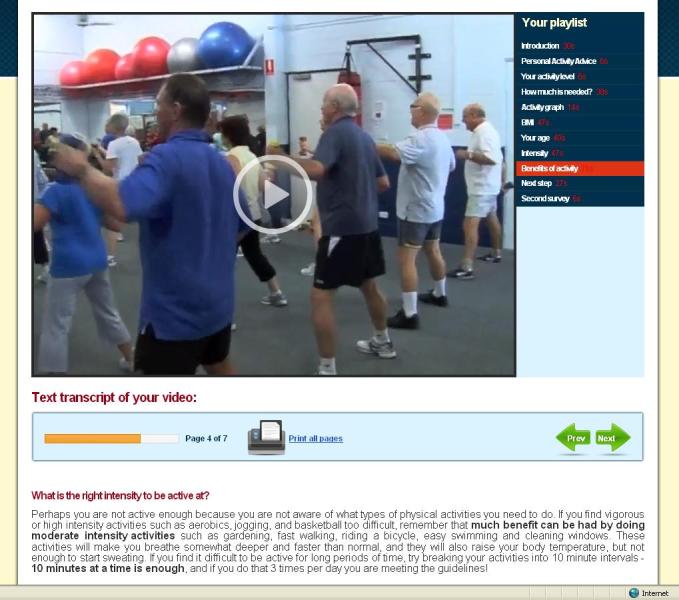
Screenshot of combination mode feedback 2.

The intervention was based on previous Internet-delivered and computer-tailored studies that successfully increased physical activity [34-38]. However, we developed additional intervention delivery modes. In addition to the previously developed text mode, we developed a video mode and a combination mode for this study. To inform the development of the video-tailored content, focus groups and a statewide survey were conducted to explore perceived appropriateness of the new delivery modes and volume of information presented [11]. The computer-tailored content of the three intervention modes was identical; only the intervention delivery mode was different. We did not change the intervention contents during the trial. A series of screenshots provides an impression of the intervention and shows the home page (Figure 2), an example of survey questions (Figure 3), an example of text mode (Figure 4); an example of video mode (Figure 5), and two examples of combination mode (Figure 6, Figure 7).

The intervention was largely based on the theory of planned behavior [39] and the stage of change concept [40]. Constructs of the theory of planned behavior were presented through provision of personally relevant feedback on attitudes, self-efficacy, intentions, benefits, and barriers in relation to their physical activity level. The intervention content was also adapted based on participants’ stage of change, and normative feedback (whether participants met the physical activity recommendation [41]) was provided in a graph. Other nontheoretical tailored variables were age, body mass index (BMI), work environment, and the distance to often-visited places. To receive personalized physical activity advice, participants first had to complete a short questionnaire about their physical activity levels, after which the personal advice immediately appeared on screen. Participants who did not meet the physical activity recommendation were encouraged to receive more feedback by completing additional questions related to the psychosocial correlates of physical activity. Participants were provided with unlimited access to the intervention website during the intervention period.

### Measures

We assessed all measures using a Web-based survey. The following demographic information was collected: gender, age, height and weight (to calculate BMI), employment status (unemployed; employed), level of education (low education being up to high school; high education being university or other tertiary degree), and confidence with using the Internet (“How confident are you with using the Internet for general purposes?”) whereby not confident at all, not confident, and neither confident nor not confident were scored as low confidence; and whereby very confident and confident were scored as high confidence.

*Physical activity level* was measured using the Active Australia Survey, which has demonstrated good validity in different population groups [42,43], as well as being sensitive to change in intervention trials [44]. Questions included items on duration and frequency of walking, and of moderate- and vigorous-intensity physical activity in the previous week. To be included, all activities had to be performed continuously for at least 10 minutes at a time.

*Preferred intervention delivery mode* was measured using the following question: “If you were going to receive personally relevant physical activity feedback via the Internet, which intervention mode of delivery would you prefer?” The answering options were “Personally relevant written text that can be read or printed out,” “A personal video message that you can watch online or download,” and “A personal video message added with transcripts that can be printed.” This question was asked during the baseline assessment (before randomization), as well as 1 week after participants received the intervention, to assess whether being exposed to a nonpreferred delivery method would change participants’ opinions about their preference.

For the assessment of *physical activity advice acceptability* and *website usability* (assessed only at the 1-week time point), surveys were largely based on previous published questionnaires [36,45,46]. Physical activity advice acceptability was assessed through 13 items on a 5-point Likert scale (from 1, strongly disagree, to 5, strongly agree) and divided (using factor analysis) into two scales. The first scale, physical activity advice content, was measured by 8 items, such as “The physical activity advice is credible” (alpha = .90). The second scale, physical activity advice layout, was assessed by 5 questions, such as “I liked the format through which the physical activity advice was provided” (alpha = .87). Website usability was measured by 22 items on a 5-point Likert scale (from 1, strongly disagree, to 5, strongly agree) and also divided into two scales. The website layout scale was assessed by 8 questions, such as “I liked the overall organization of the website—the links, tabs, and buttons” (alpha = .92), and the website ease of use scale was measured by another 14 questions, such as “I was able to easily find my way around the website” (alpha = .94).

*Website user statistics*, measuring time spent on website, were collected during the entire 1-month intervention period.

### Analysis

We created two groups based on whether the intervention delivery mode to which participants were assigned was matched or mismatched with their preferred intervention delivery mode. For example, participants were mismatched if they were assigned to receive the intervention in video mode, yet they preferred to receive the intervention in text mode. Vice versa, participants were matched if, for example, they preferred to receive the intervention in combination mode (video and text based) and were actually also randomly assigned to this group.

We used 1-way analyses of variance, independent-samples *t* tests, and chi-square tests to analyze dropout, compare baseline characteristics, and examine differences in website usability, physical activity advice acceptability, and time spent on the intervention website between the matched and mismatched groups.

According to the Active Australia Survey guidelines for analysis and reporting, we computed total physical activity minutes by summing the time spent walking and on moderate- and vigorous-intensity physical activity in the past week (vigorous-intensity physical activity was weighted by two) [47]. Walking, moderate-intensity physical activity, and vigorous-intensity physical activity were added together for the total number of physical activity sessions of the past week. To account for overreporting, we truncated each activity type on the Active Australia Survey at 14 hours per week, and total activity at a maximum of 28 hours per week. A sufficient level of physical activity was defined as being active for at least 150 minutes spread across a minimum of 5 sessions each week [47].

To evaluate the intervention effects on physical activity, we conducted repeated-measures analyses of covariance with time (baseline, 1 week, 1 month) as the within-participants factor and group (matched and mismatched) as the between-participants factor, controlled for baseline differences and delivery mode to which participants were assigned. We used both an intention-to-treat analysis (last value carry forward; n = 803) and a retained-sample analysis (n = 288). All analyses were performed using SPSS version 18.0 (IBM Corporation, Somers, NY, USA). Statistical significance was set at a level of .05.

## Results

### Participants

Of 3233 people contacted, 1332 (41.20%) completed the PAR-Q screening. Of these, we excluded 450 (33.8%) because they were not eligible according to the screening questionnaire. Of the 863 participants who completed the baseline questionnaire, we excluded from further analysis 51 (6%) because they did not receive any tailored feedback and 9 (1%) because they did not visit the website, yielding 803 participants. The 1-week follow-up questionnaire was completed by 370 (42.9%) respondents, and 288 (33.4%) completed the 1-month follow-up (see Figure 1).

Table 1 shows participant characteristics at baseline. The average age of all respondents was 52.4 years (range 19–89 years) and the majority were women (60.7%). Levels of education (78.5%), employment (70.9%), and Internet confidence (84.3%) were high. No significant baseline differences were observed in any of the examined variables between participants in the matched and mismatched groups, except for the intervention mode to which participants were originally randomly assigned (χ^2^_2,801_ = 151.3, *P* < .001). This was because preferences were distributed differently for each delivery mode.

**Table 1:** Participant characteristics at baseline across matched and mismatched groups.

		Total study population (n = 803)	Group^a^	
			Match (n = 261)	Mismatch (n = 542)
**Gender, n(%)**				
	Male	316 (39.4%)	103 (39.5%)	213 (39.3%)
	Female	487 (60.7%)	158 (60.5%)	329 (60.7%)
Age (years), mean (SD)		52.4 (11.9)	51.6 (12.3)	52.8 (11.8)
BMI^b^ (kg/m^2^), mean (SD)		27.3 (6.2)	26.7 (5.3)	27.6 (6.7)
**Employment status, n(%)**				
	Unemployed	234 (29.1%)	77 (30%)	157 (29.0%)
	Employed	569 (70.9%)	184 (70.5%)	385 (71.0%)
**Internet confidence, (n, %)**				
	Low	126 (15.7%)	37 (14%)	89 (16%)
	High	677 (84.3%)	224 (85.8%)	453 (83.6%)
**Education level, n (%)**				
	Low	173 (21.5%)	57 (22%)	116 (21.4%)
	High	630 (78.5%)	204 (78.6%)	426 (78.6%)
**Baseline physical activity level, n (%)**				
	Insufficient	352 (43.8%)	109 (41.8%)	243 (44.8%)
	Sufficient	451 (56.2%)	152 (58.2%)	299 (55.2%)
**Intervention condition, n (%)**				
	Video based	258 (32.1%)	31 (12%)	227 (41.9%)
	Text based	262 (32.6%)	159 (60.9%)	103 (19.0%)
	Combination	283 (35.2%)	71 (27%)	212 (39.1%)

^a^ No baseline differences were observed between matched and mismatched participants, except for intervention condition (χ^2^_2,801_= 151.3, *P* < .001).

^b^ Body mass index.

#### Dropout

Dropout levels differed only for the age of participants. Young people were significantly more likely than older participants to drop out during the course of the study (*t*_86__3_ = 4.23, *P* = .000); the mean age of dropouts (50.8 years) was lower than the mean age of completers (54.3 years). No differences in dropout levels were observed for any other variables. Specifically, when comparing matched and mismatched participants, dropout was somewhat higher in the matched group (188/281, 66.9%) than in the mismatched group (387/582, 66.4%), but this difference was not significant (*t*_86__3_ = 1.31, *P* = .19). In relation to this, few participants took the opportunity to return to the website more than once, and there were no significant differences between matched (8/194, 4%) and mismatched (17/429, 4%) groups (*t*_621_ = .65, *P* = .51).

#### Preferred Intervention Delivery Mode

Table 2 shows the distributions of delivery mode preferences between matched and mismatched groups before and after the intervention. The text mode was by far the most popular preference, the video mode was the least popular preference, and the combination mode was in the middle. At baseline, delivery mode preferences were equal between matched and mismatched participants (χ^2^_2__,801_ = 0.03, *P* = .98). After receiving the intervention, participants’ overall delivery mode preferences changed, although this was not statistically significant (χ^2^_2__,412_ = 4.1, *P* = .13).

**Table 2:** Delivery mode preferences for matched and mismatched participants pre- and postintervention.

		Video n (%)	Text n (%)	Combination n (%)	χ^2^	df	*P* value
**Preintervention**							
	Matched	31 (12%)	159 (60.9%)	71 (27%)			
	Mismatched	63 (12%)	334 (61.6%)	145 (26.8%)	0.03	2,801	.98
**Postintervention**							
	Matched	14 (11%)	92 (70%)	26 (20%)			
	Mismatched	44 (16%)	168 (59.6%)	70 (25%)	4.1	2,412	.13

While the overall proportions of participants’ preferences changed little after receiving the intervention, further examination of change in preference in the matched and mismatched groups revealed that change in preference appeared to be group dependent. After having received the intervention, 20% (26/132) of the participants in the matched group changed their delivery mode preference: this was 31% (4/13) for those who preferred the video mode at baseline, 3% (2/77) for those who preferred text mode, and 48% (20/42) for those who preferred the combination mode. In the mismatched group, 34% (96/282) of participants changed their delivery mode preference: this was 61% (17/28) for those who preferred the video mode at baseline, 25% (44/175) for those who preferred text mode, and 44% (35/79) for those who preferred the combination mode.

### Physical Activity Changes

Table 3 reports the outcomes of the repeated-measures analyses of covariance. Significant time effects were observed for all the different physical activity outcomes (total minutes of physical activity, total number of activity sessions, walking minutes, and minutes of moderate and vigorous physical activity) according to the intention-to-treat analysis; indicating that physical activity increased over time for both matched and mismatched groups combined. This was also the case for the analysis including only participants who completed all measurements, though the *F* values are lower and not all values are significant. The participants in the matched group improved physical activity outcomes slightly more than did those in the mismatched group, but we observed no significant interaction effects for either the intention-to-treat or completer analysis. Intervention effects for participants not meeting the physical activity recommendations were also calculated; outcomes were similar to those of the total group, though time effects were stronger (the *F* values ranged between 6 and 24). However, we again noted no interaction effects (outcomes not reported in table). Finally, both the matched and the mismatched groups had an increase of 5% of participants meeting the physical activity guidelines from baseline to 1 week and then a decrease of 2% from 1 week to 1 month; there were no significant differences between groups.

**Table 3:** Main and interaction effects for physical activity (mean, SD) between matched and mismatched groups.

		Intention-to-treat (n = 803)				Completers (n = 288)			
		Matched (n = 261)	Mismatched (n = 542)	Time (*F*_2,801_)	group × time (*F*_2,801_)	Matched (n = 93)	Mismatched (n = 195)	Time (*F*_2,284_)	group × time (*F*_2,284_)
**Total physical activity (minutes)**									
	Baseline	336 (352)	315 (342)			308 (301)	314 (344)		
	1 week	355 (339)	320 (325)			348 (265)	322 (293)		
	1 month	362 (362)	335 (345)	5.07**	0.87	355 (332)	344 (339)	2.16	0.59
	Difference	+26	+20			+47	+30		
**Total physical activity sessions**									
	Baseline	9.7 (7.1)	9.9 (9.3)			9.0 (6.1)	9.5 (7.5)		
	1 week	10.2 (7.1)	10.3 (9.8)			9.6 (5.7)	10.3 (8.9)		
	1 month	10.5 (7.6)	10.6 (9.4)	7.52***	0.04	10.1 (7.4)	11.2 (8.7)	5.63**	0.23
	Difference	+0.8	+0.7			+1.1	+1.7		
**Walking (minutes)**									
	Baseline	146 (158)	134 (154)			129 (146)	142 (167)		
	1 week	153 (155)	139 (150)			141 (139)	143 (140)		
	1 month	162 (159)	148 (159)	8.32***	0.08	165 (156)	154 (161)	4.71*	0.93
	Difference	+16	+14			+36	+12		
**Moderate-intensity activity (minutes)**									
	Baseline	54 (116)	50 (121)			44 (83)	54 (142)		
	1 week	66 (125)	56 (110)			55 (102)	56 (107)		
	1 month	70 (132)	59 (111)	9.53***	1.13	56 (122)	60 (111)	0.49	0.19
	Difference	+16	+9			+12	+6		
**Vigorous-intensity activity (minutes)**									
	Baseline	69 (121)	69 (130)			66 (111)	60 (105)		
	1 week	76 (118)	73 (125)			75 (102)	66 (113)		
	1 month	80 (125)	79 (133)	6.04**	0.26	67 (116)	66 (122)	0.64	0.22
	Difference	+11	+10			+1	+6		

* *P* < .05, **P < .01, ***P < .001.

### Physical Activity Advice Acceptability, Website Usability, and Time Spent on the Website

Physical activity advice acceptability and website usability were generally high, and differences between the matched and mismatched groups were few (Table 4). The only significant difference (*t*_621_ = 2.16, *P* = .03) was in relation to the total time spent on the website: those in the mismatched group spent significantly more time on the website (14.4 minutes) than those in the matched group (12.1 minutes). Thus, exposure to intervention materials in the mismatched group was significantly greater than in the matched group.

**Table 4:** Differences between matched and mismatched groups for acceptability of activity advice, website usability, and time spent on the website (mean, SD).

		Total study population (n = 428)	Matched (n = 137)	Mismatched (n = 291)	*t* test	df
**Physical activity advice acceptability**^a^						
	Advice content	3.2 (0.7)	3.2 (0.6)	3.2 (0.7)	0.10	368
	Advice layout	3.9 (0.5)	3.8 (0.5)	3.9 (0.5)	1.53	368
**Website usability**^a^						
	Layout	3.7 (0.5)	3.7 (0.5)	3.7 (0.6)	0.05	426
	Ease of use	4.0 (0.5)	4.0 (0.5)	4.0 (0.5)	0.21	426
Time spent on website (minutes)		13.7 (12.2)	12.1 (9.6)	14.4 (13.1)	2.16^b^	621

^a^ On a scale ranging from 1, strongly disagree, to 5, strongly agree.

^b^ *P* = .31.

## Discussion

The main finding of this study was that delivery mode preference does not influence behavioral outcomes and other outcomes that are important in the effectiveness of Web-based interventions. The acceptability, usability, and effectiveness of the physical activity intervention was not significantly different for participants matched or mismatched to their preferred intervention delivery mode (video based, text based, or combination). This finding is in line with other studies that have examined preference effects for other types of interventions within different populations [18-20,48]. Only in their study on human papillomavirus testing did McCaffery et al [30] find effects on quality of life based on preferences for different interventions. The outcomes of the current study confirm the conclusion by King et al [19]: although participants may have strong intervention preferences, there is not much support for the hypothesis that preferences significantly compromise the internal validity of randomized controlled trials. The outcomes indicate that health promotion practitioners can be guided by efficacy outcomes obtained through randomized controlled trials and do not have to accommodate participant preferences with regard to intervention delivery modes in Web-based, computer-tailored physical activity interventions, as doing so would not increase intervention effectiveness.

It is difficult to explain why no differences were found, as the findings seem counterintuitive [18]. It might be that the differences between the intervention delivery modes in this study were too small to alter effectiveness. Preferences may have more impact when, for example, comparing a face-to-face intervention with a website-delivered intervention [25,26]. Different variations of the same website-delivered intervention may have been too subtle to be influential. Or a form of recruitment bias might have been at play. As all participants were reactively recruited via email, it might be that the participants liked interacting with any kind of technology or delivery mode, thus providing little opportunity for the influence of preference. This is in line with the high levels of Internet confidence reported. Alternatively, Lewis et al [18] suggested that perhaps, after being randomly assigned to a nonpreferred intervention, participants are pleasantly surprised regarding the components of the intervention and care less about being assigned to the a nonpreferred intervention. This is in line with King’s [21] assumption that the stability of attitudes will affect the internal validity; a positive experience with an intervention during a trial may change negative attitudes and weaken the effect of preference on outcomes.

Though acceptability of the advice and website usability were not influenced by delivery mode preferences in this study, others have seen an impact of participant satisfaction levels [20]. Therefore, in line with Foley et al [49], we recommend offering participants a delivery mode choice if possible, as it will not harm intervention effectiveness, and might also increase participant satisfaction levels. The current study unexpectedly found that mismatched participants spend significantly more time on the website, yet this longer exposure to intervention materials did not translate to differences in outcomes. This is in contrast to previous studies that have emphasized the importance of intervention exposure to achieve behavioral change [50-52]. However, while the differences in time spent on the website were statistically significant, we do not know whether this difference in time (approximately 2.3 minutes) was meaningful enough to alter behavior. Alternatively, it might be that the extra time spent on the website was of low quality in relation to paying attention to the physical activity message. Participants might have been distracted by emails or might have visited another website simultaneously. As the online environment is extremely competitive, further research should investigate the effects on outcomes of paying attention to website-delivered interventions.

When examining the preferences themselves, we noted that strong support for the text-based delivery mode was evident, and this remained so after participants were exposed to the text mode. Also, change in preference was very low among those who preferred and received a text mode intervention ((2/77, 3%), and it was very high for those who preferred a video mode intervention but did not receive it (17/28, 60%). This is in contrast with eye-tracking research, which has shown that people don’t fully read text on the Internet; instead, they scan and skim the content [53,54]. Internet-based reading behavior is characterized by more time spent on browsing and scanning, keyword spotting, nonlinear reading, and more selective reading, while less time is spent on in-depth and concentrated reading [53,55]. Perhaps this is different when the information offered is personally relevant, as is the case with computer-tailored interventions [12]. According to the Elaboration Likelihood Model, information is processed more thoroughly when it is perceived to be personally relevant [56]. Finally, change in delivery mode preferences, after participants received the intervention, was greatest in the mismatched group (34.0% vs 19.7%). This was not unexpected, as for matched participants a change in preference is likely related to dissatisfaction with their original choice; for mismatched participants a change in preference is likely related to satisfaction with the delivery mode to which they were exposed during the intervention over their original preference.

This study has some limitations that limit its generalizability. First, the low-intensity real-life implementation of the current study (email recruitment, no face-to-face or telephone contact for the entire study) was more than likely responsible for the high attrition levels [57,58]. Yet these dropout levels are comparable with those of other website-delivered studies with similar protocols [59-61]. Second, as also mentioned by Lewis et al [18], participants were administered a forced-choice question regarding preference. In other words, even when participants didn’t have a preference, or would have preferred not to use any of the delivery modes presented to them, they were forced to make a choice. In relation to this, participants might have had a different preference if they would have been able to experience each of the delivery modes beforehand; due to the innovative nature of this intervention and its delivery modes (with which the participant would have been unfamiliar), this might have indeed been the case. However, from a practical point of view, it was not possible to expose participants to the delivery mode options prior to the randomized trial. A third limitation is that the sample was relatively homogeneous (eg, mostly white and educated). The effect of preference may possibly vary across sociodemographic variables. Fourth, a larger sample size is needed to explore the effects of preference within each intervention delivery mode (video based, text based, and combination) separately; the current study was not sufficiently powered to do so. Fifth, the follow-up period in this study was short, and potentially preferences play a greater role in maintenance and use of different delivery modes over the longer term.

In conclusion, this study illustrates that the importance of preference effects in different delivery modes of an Internet-based physical activity intervention is limited. However, due to the scarcity of research in this area, more studies to investigate this research topic that can address the above-mentioned limitations are needed.

## Multimedia Appendix 1

CONSORT-EHEALTH form V1.6 [62].

## Abbreviations

BMI: body mass index
PAR-Q: Physical Activity Readiness Questionnaire
