# Supplementary material for: Do Participants’ Preferences for Mode of Delivery (Text, Video, or Both) Influence the Effectiveness of a Web-Based Physical Activity Intervention?
Source: J Med Internet Res. 2012 Feb 29;14(1):e37. doi: 10.2196/jmir.1998 (PMC3374539; doi:10.2196/jmir.1998)
Supplement: Supplementary file 1 [file jmir_v14i1e37_app1.pdf]

CONSORT-EHEALTH Checklist V1.6 Report

**Manuscript Number**	1998
**Date completed**	8/25/2011 14:50:29
**by**	Corneel Vandelanotte

Do participants' preferences for mode of delivery (text, video or both) influence the effectiveness of an web-based physical activity intervention?

TITLE

1a-i) Identify the mode of delivery in the title

Do participants' preferences for mode of delivery (text, video or both) influence the effectiveness of a web-based physical activity intervention?

1a-ii) Non-web-based components or important co-interventions in title

Not applicable, there were no non-webased components or co-interventions.

1a-iii) Primary condition or target group in the title

Not applicable, study participants were adults from the general population

ABSTRACT

1b-i) Key features/functionalities/components of the intervention and comparator in the METHODS section of the ABSTRACT

YES, "Adults (n=803), recruited via e-mail, were randomized into one of three modes of intervention delivery (text, video, combined) and received fully automated personal advice about physical activity via the internet. Intervention content was identical across groups."

1b-ii) Level of human involvement in the METHODS section of the ABSTRACT

YES, "Adults (n=803), recruited via e-mail, were randomized into one of three modes of intervention delivery (text, video, combined) and received fully automated personal advice about physical activity via the internet. "

1b-iii) Open vs. closed, web-based (self-assessment) vs. face-to-face assessments in the METHODS section of the ABSTRACT

YES,
"Adults (n=803), recruited via e-mail, were randomized into one of three modes of intervention delivery (text, video, combined) and received fully automated personal advice about physical activity via the internet. Intervention content was identical across groups."

"Self-assessed questionnaires were completed at baseline, one week and one month."

1b-iv) RESULTS section in abstract must contain use data

YES,
"Attrition was high (67%); 93 participants were categorised as 'matched' and 195 as 'mismatched'."

1b-v) CONCLUSIONS/DISCUSSION in abstract for negative trials

Not applicable, though participants' preference had little influcne on intervention outcomes, physical activity levels did increase accross groups during the intervention.

INTRODUCTION

2a-i) Problem and the type of system/solution

YES,
Page 3, first paragraph: "Physical inactivity increases the risk of developing cardiovascular disease, diabetes, hypertension, some cancers and obesity [1,2]. As large proportions of the population are not meeting physical activity guidelines [3-5], increasing physical activity is a public health priority. As such, intervention strategies that can reach many people in a cost-effective manner are desired."

2a-ii) Scientific background, rationale: What is known about the (type of) system

YES,
Page 3, second paragraph:"Web-based physical activity interventions have shown promising results [6-8] and will continue to gain importance through growth in internet access (in Australia 62% of households has broadband access), power of web-based applications (e.g. Facebook, YouTube) and convenience (through mobile devices) [9,10]. However, the immense versatility of the internet allows for health information to be delivered in several ways [8]. For example, interventions delivered via websites can provide personally tailored information through different modes, such as text-based, video-based or both [11]. Whilst personally tailored interventions have shown to be efficacious both in offline (print-based) and online studies [12-15], there are large variations in individual preferences for the mode of intervention delivery [16-17]. This raises the question as to whether the efficacy of an intervention may be enhanced or reduced when it is provided through a preferred or non-preferred mode [18]. "

METHODS

3a) CONSORT

YES,
Page 4, second paragraph:"Therefore, this study aims to examine the impact of receiving computer-tailored intervention content that is either matched or mismatched with participants' preferred mode of delivery (text-based, video-based or both) on the acceptability, usability and effectiveness of a web-based physical activity intervention. "

3b-i) Bug fixes, Downtimes, Content Changes

Not applicable

4a-i) Computer / Internet literacy

Not explicitly: Though all participants would have had some form of Internet literacy (Internet access was an eligiblity criteria, and all measures were web-based), the intervention that was evaluated was designed as such that only minimal Internet literacy would have been needed in order to receive it. Therefore it Internet literacy was not an eligibility criterion in this study.

4a-ii) Open vs. closed, web-based vs. face-to-face assessments:

YES,
Page 4, forth paragraph:"In January and February 2011, male and female adults over 18 years of age from the general population in Australia were invited by e-mail to participate in the study. People listed in a database held by the Population Research Laboratory at the Institute of Health and Social Sciences Research at the University of Central Queensland were invited. "

Page 4, forth paragraph:"The whole study was entirely web-based without any face-to-face components as part of the intervention or the assessment; as such 'real-life' conditions were mimicked as closely as possible. "

4a-iii) Information giving during recruitment

YES,
Page 4, forth paragraph:"The invitation e-mails contained a link to a website with information about the nature and purpose of the present study and access to the baseline survey. By accessing the baseline survey, participants provided consent to participate and agreed that they were well informed about the study."

4b-i) Report if outcomes were (self-)assessed through online questionnaires

YES:
Page 5, paragraph 4: "All measures were assessed using a web-based survey. "

4b-ii) Report how institutional affiliations are displayed

This is not reported in the paper: The invitation e-mail clrealy indicated they would be participating in research conducted by researchers at the Central Queensland University. The survey and intervention websites had logo's of the Central Queensland University. The home page of the intervention website also mentions the principal research who developed the website and his affiliation.

5-i) Mention names, credential, affiliations of the developers, sponsors, and owners

YES, though it is not explicitly mentioned. By looking up the authors on the references mentioned below, it becomes obvious that the authors/evaluators are also the owners/developpers of the software.
Page 5, paragraph 2: "The intervention was based on previous internet-delivered and computer-tailored studies that successfully increased physical activity [34-38], however additional intervention delivery-modes were developed. In addition to the previously developed text-mode, a video-mode and a combination-mode (video- and text-based) were developed for the purpose of this study. To inform the development of the video-tailored content focus groups and a state-wide survey were conducted to explore perceived appropriateness of the new delivery modes and volume of information presented [11]. "

5-ii) Describe the history/development process

YES,
Page 5, paragraph 2: "The intervention was based on previous internet-delivered and computer-tailored studies that successfully increased physical activity [34-38], however additional intervention delivery-modes were developed. In addition to the previously developed text-mode, a video-mode and a combination-mode (video- and text-based) were developed for the purpose of this study. To inform the development of the video-tailored content focus groups and a state-wide survey were conducted to explore perceived appropriateness of the new delivery modes and volume of information presented [11]. "

5-iii) Revisions and updating

YES,
Page 5, paragraph 2: "The intervention was based on previous internet-delivered and computer-tailored studies that successfully increased physical activity [34-38], however additional intervention delivery-modes were developed. In addition to the previously developed text-mode, a video-mode and a combination-mode (video- and text-based) were developed for the purpose of this study. To inform the development of the video-tailored content focus groups and a state-wide survey were conducted to explore perceived appropriateness of the new delivery modes and volume of information presented [11]. The computer-tailored content of the three intervention-modes were identical, only the intervention delivery-mode was different. No changes were made to the intervention contents during the trial. "

5-iv) Quality assurance methods

YES,
Page 5, paragraph 2: "To inform the development of the video-tailored content focus groups and a state-wide survey were conducted to explore perceived appropriateness of the new delivery modes and volume of information presented [11].

5-v) Ensure replicability by publishing the source code, and/or providing screenshots/screen-capture video, and/or providing flowcharts of the algorithms used

YES, screenshots illustrating the different delivery modes are provided in the manuscript.

5-vi) Digital preservation

YES, screenshots illustrating the different delivery modes are provided in the manuscript.

5-vii) Access

YES,
Page 4, paragraph 4: "After completing the baseline survey, a link to the intervention website was provided; participants were automatically randomised into one of the three groups upon accessing the website. The whole study was entirely web-based without any face-to-face components as part of the intervention or the assessment; as such 'real-life' conditions were mimicked as closely as possible. "

5-viii) Mode of delivery, features/functionalities/components of the intervention and comparator, and the theoretical framework

YES:
Page 5, paragraphs 2 and 3:" The intervention was based on previous internet-delivered and computer-tailored studies that successfully increased physical activity [34-38], however additional intervention delivery-modes were developed. In addition to the previously developed text-mode, a video-mode and a combination-mode (video- and text-based) were developed for the purpose of this study. To inform the development of the video-tailored content focus groups and a state-wide survey were conducted to explore perceived appropriateness of the new delivery modes and volume of information presented [11]. The computer-tailored content of the three intervention-modes were identical, only the intervention delivery-mode was different. No changes were made to the intervention contents during the trial.

The intervention was largely based on the Theory of Planned Behavior [39] and the Stage of Change concept [40]. Constructs of the Theory of Planned Behavior were presented through provision of personally relevant feedback on attitudes, self-efficacy, intentions, benefits and barriers in relation to their physical activity level. The intervention content was also adapted based on participants' stage of change and 'normative feedback' (whether or not participants meet the physical activity recommendation [41]) was provided in a graph. In order to receive personalized physical activity advice, participants first had to complete a short questionnaire about their physical activity levels, after which the personal advice immediately appeared on screen. Participants who did not meet the physical activity recommendation were encouraged to receive more feedback by completing additional questions related to the psychosocial correlates of physical activity. Participants were provided with unlimited access to the intervention website during the intervention period."

5-ix) Describe use parameters

YES,
Page 5, paragraphs 3: "In order to receive personalized physical activity advice, participants first had to complete a short questionnaire about their physical activity levels, after which the personal advice immediately appeared on screen. Participants who did not meet the physical activity recommendation were encouraged to receive more feedback by completing additional questions related to the psychosocial correlates of physical activity. Participants were provided with unlimited access to the intervention website during the intervention period."

5-x) Clarify the level of human involvement

YES,
Page 5, Paragraph 1: "The whole study was entirely web-based without any face-to-face components as part of the intervention or the assessment; as such 'real-life' conditions were mimicked as closely as possible. "

5-xi) Report any prompts/reminders used

YES,
Page 5, paragraph 1: "Non-responders were reminded three times to complete each assessment."

5-xii) Describe any co-interventions (incl. training/support)

Not applicable, there were no co-interventions.

6a-i) Online questionnaires: describe if they were validated for online use and apply CHERRIES items to describe how the questionnaires were designed/deployed

Though the survey's were not validated for online use, they have been used in online studies many times without problems being reported.

6a-ii) Describe whether and how "use" (including intensity of use/dosage) was defined/measured/monitored

YES,
"Website user statistics, measuring 'time spent on website', were collected during the entire one month intervention period. "

6a-iii) Describe whether, how, and when qualitative feedback from participants was obtained

Participants had the option to provide feedback to the researchers (and ethics committee) via e-mail and a form on the website; it was rarely used and not worth reporting on in the manuscript.

7a-i) Describe whether and how expected attrition was taken into account when calculating the sample size

No sample size calculations were conducted, due to our methodology for this pilot study we had access to a fixed number of e-mail addressess, we tried to recruit as much participants as possible. Despite the high attrittion (which was expected), the sample obtained was large enough to perform the secondary analyses presented in the current manuscript.

7b) CONSORT

Not applicable.

8a) CONSORT

YES,
Page 4, paragraph 4: " participants were automatically randomised into one of the three groups upon accessing the website"

Page 6, second paragraph: Preferred intervention delivery mode question: "This question was asked during the baseline assessment (before randomization)"

Page 6, 5th paragraph: "Two groups were created based on whether the intervention delivery-mode to which participants were randomized was 'matched' or 'mismatched' with their preferred intervention delivery-mode. For example, participants were 'mismatched' if they were randomized to receive the intervention in video-mode, yet they preferred to receive the intervention in text-mode. Vice versa, participants were 'matched' if, for example, they preferred to receive the intervention in combination-mode (video and text) and were actually also randomized to this group. "

8b) CONSORT

YES,
Page 4, paragraph 4: " participants were automatically randomised into one of the three groups upon accessing the website"

9) CONSORT

YES,
Page 4, paragraph 4: " participants were automatically randomised into one of the three groups upon accessing the website"

10) CONSORT

YES,
Page 4, paragraph 4: " participants were automatically randomised into one of the three groups upon accessing the website"

11a-i) Specify who was blinded, and who wasn't

Nobody was blinded, as randomisation occurred automatically via the website, and all contact with participants was through automated reminder e-mails and online surveys, there was no need to blind anyone, as there was no opportunity to influence participants in any kind of way. Given this methodology, the issue of blinding was not mentionned in the manuscript.

11a-ii) Discuss e.g., whether participants knew which intervention was the "intervention of interest" and which one was the "comparator"

Not applicable: as this study reports on a 'randomised trial', not a 'randomised controlled trial', all interventions were of interest, and all were compared with each other. No information was concealed, the participants were aware of the interventions they did not receive.

11b) CONSORT

YES,
Page 5, paragraph 2: "In addition to the previously developed text-mode, a video-mode and a combination-mode (video- and text-based) were developed for the purpose of this study. The computer-tailored content of the three intervention-modes were identical, only the intervention delivery-mode was different. "

12a) CONSORT

YES,
Page 6, paragraph 6:" One way ANOVAs, independent samples t-tests and Chi2-tests were used for analysing drop-out, comparing baseline characteristics, and to examine differences between website usability, physical activity advice acceptability and time spent on the intervention website between the matched and mismatched groups.

To evaluate the intervention effects on physical activity, repeated measures ANCOVAs with time (baseline, one week, one month) as within-subjects factor and group (matched and mismatched) as between-subjects factors, controlled for baseline differences and delivery-mode to which participants were randomised, were conducted using both an intent-to-treat analysis (last value carry forward; n = 803) and a retained sample analysis (n = 288). All analyses were performed using SPSS 18.0. Statistical significance was set at a level of .05."

12a-i) Imputation techniques to deal with attrition / missing values

YES,
Page 7, second paragraph: "To evaluate the intervention effects on physical activity, repeated measures ANCOVAs with time (baseline, one week, one month) as within-subjects factor and group (matched and mismatched) as between-subjects factors, controlled for baseline differences and delivery-mode to which participants were randomised, were conducted using both an intent-to-treat analysis (last value carry forward; n = 803) and a retained sample analysis (n = 288). "

12b) CONSORT

YES,
Page 7, second paragraph: "To evaluate the intervention effects on physical activity, repeated measures ANCOVAs with time (baseline, one week, one month) as within-subjects factor and group (matched and mismatched) as between-subjects factors, controlled for baseline differences and delivery-mode to which participants were randomised, were conducted using both an intent-to-treat analysis (last value carry forward; n = 803) and a retained sample analysis (n = 288). "

RESULTS

13a) CONSORT

YES, see Figure 1, the flowchart (on page 8). The tables also indicate the number of participants analysed for each outcome.

13b) CONSORT

YES, see Figure 1, the flowchart (on page 8).

13b-i) Attrition diagram

Not applicable, the duration of the intervention was very short and the number of people that visited the website more than once extremely low. Rather than an attrition diagram, total time participants spend on the website for each group was reported.

14a) CONSORT

YES,
Page 4, paragraph 4: "In January and February 2011, male and female adults over 18 years of age from the general population in Australia were invited by e-mail to participate in the study. "

14a-i) Indicate if critical "secular events" fell into the study period

Not applicable, the intervention duration was very short in any case.

14b) CONSORT

Not applicable.

15) CONSORT

YES, Table 1 (on page 9) provides participant characteristics at baseline for total group and for intervention groups.

15-i) Report demographics associated with digital divide issues

YES, the following variables were reported in table 1 (on page 9): gender, age, BMI, Employment status, educational level and internet confidence.

16-i) Report multiple "denominators" and provide definitions

YES, N values were reported througout the results section, in all tables, for all analyses and in the flow chart.

16-ii) Primary analysis should be intent-to-treat

YES, intention-to-treat was applied and reported as primary analysis (Table 3 page 11).

17a) CONSORT

YES, test (e.g. F-value, t-value) and P values were reported where applicable in the result section.

17a-i) Presentation of process outcomes such as metrics of use and intensity of use

YES, time spent on website was reported in table 4 (on page 12).

17b) CONSORT

YES, this was done in tables 1 (on page 9) and 2 (on page 10).

18) CONSORT

Not applicable.

18-i) Subgroup analysis of comparing only users

YES, this is only relevant to table 3 (on page 11), where both intention-to-treat as well as a completer analysis are presented.

19) CONSORT

Not applicable, no harms were reported to the investigators.

19-i) Include privacy breaches, technical problems

Not applicable, no privacy breaches or technical problems were reported to the investigators.

19-ii) Include qualitative feedback from participants or observations from staff/researchers

Not available.

DISCUSSION

20-i) Typical limitations in ehealth trials

YES,
Page 13, paragraph 4: "This study has a number of limitations that limit its generalizability. First, the low intensity 'real-life' implementation of the current study (e-mail recruitment, no face-to-face or telephone contact for the entire study) is more than likely responsible for the high attrition levels [56,57]. Yet these drop-out levels are comparable to those of other website-delivered studies with similar protocols [58-60]. Second, as also mentioned by Lewis et al. (2006), participants were administered a forced choice question regarding preference [18]. In other words, even when participants didn't have a preference they were forced to make a choice. In relation to this, participants might have had a different preference if they would have been able to experience each of the delivery modes beforehand; due to the innovative nature of this intervention and its delivery-modes (to which the participant would have been unfamiliar) this might have indeed been the case. Though, from a practical point of view it was not possible to expose participants to the delivery-mode options prior to the randomised trial. Third, a further limitation is that the sample was relatively homogenous (e.g., mostly Caucasian, educated,...). It is possible that the effect of preference may vary across socio-demographic variables. Forth, a larger sample size is needed to explore the effects of preference within each intervention delivery-mode (video, text, combination) separately; the current study was not sufficiently powered to do so. "

21-i) Generalizability to other populations

YES,
Page 5, paragraph 1: "The whole study was entirely web-based without any face-to-face components as part of the intervention or the assessment; as such 'real-life' conditions were mimicked as closely as possible."

21-ii) Discuss if there were elements in the RCT that would be different in a routine application setting

YES,
Page 5, paragraph 1: "The whole study was entirely web-based without any face-to-face components as part of the intervention or the assessment; as such 'real-life' conditions were mimicked as closely as possible."

22-i) Restate study questions and summarize the answers suggested by the data, starting with primary outcomes and process outcomes (use)

YES,
Page 12, paragraph 1:"The main finding of this study is that delivery-mode preference does not influence behavioural outcomes and other outcomes that are important in the efficacy of web-based interventions; the acceptability, usability and effectiveness of the physical activity intervention was not significantly different for participants matched or mismatched to their preferred intervention delivery-mode (video, text or combination)."

22-ii) Highlight unanswered new questions, suggest future research

YES,
Page 12, second parageaph: "As the online environment is extremely competitive, further research should investigate the effects of providing 'attention' to website-delivered interventions on outcomes. "

Page 14, second paragraph: "In conclusion, this study illustrates that the importance of preference effects in different delivery-modes of an internet-based physical activity intervention is limited; however due to the scarcity of research in this area, more studies to investigate this research topic, which can address the above mentioned limitations, are needed."

Other information

23) CONSORT

No, initially this study was set up as a pilot study, as a precursor to a larger more intensive intervention study. This study was only to create 'preliminary data' to support grant proposals. As such, the trial was not registered. However, given the number of participants, we were able to answer research questions interesting and relevant enough for publication. The main aim of traily registry (to prevent the selective reporting of trial outcomes or withoulding of data) is thus not relevant for this manuscript.

24) CONSORT

In combination with the references provided trhoughout the manuscript all relevant protocol information has been presented, avoiding the need to provided additional protocol information elsewhere.

25) CONSORT

YES,
Page 14, third paragraph: "This study was funded by a Central Queensland University Merit Grant (which is part of the Research Development and Incentive Program). The Population Research Laboratory (PRL), managed by Ms Christine Hanley, was responsible for participant recruitment and data collection using web-based surveys. Dr Vandelanotte was supported by a National Health and Medical Research Council of Australia (#519778) and National Heart Foundation of Australia (#PH 07B 3303) post-doctoral research fellowship. Professor Plotnikoff was supported by a Senior Research Fellowship from the National Health and Medical Research Council of Australia."

X26-i) Comment on ethics committee approval

YES,
Page 5, first paragraph: "The study received ethical approval by the Human Research Ethics Committees at Central Queensland University."

x26-ii) Outline informed consent procedures

YES,
Page 4, paragraph 4: "By accessing the baseline survey, participants provided consent to participate and agreed that they were well informed about the study. "

X26-iii) Safety and security procedures

YES,
Page 4, paragraph 4: "The Physical Activity Readiness Questionnaire (PAR-Q) was used to screen for participants for whom it was not safe to increase physical activity [33]. If participants answered 'yes' to one of the PAR-Q questions they were thanked for their time and not provided with access to the intervention website."

X27-i) State the relation of the study team towards the system being evaluated

YES,
Page 14, 4th paragraph: "Dr Vandelanotte is the owner and co-developer of the intervention presented in this study. There are no other conflicts of interest. "
